# Association between arterial tortuosity and early neurological deterioration in lenticulostriate artery infarction

**DOI:** 10.1038/s41598-023-47281-8

**Published:** 2023-11-14

**Authors:** Sang Hee Ha, Soo Jeong, Jae Young Park, Jun Young Chang, Dong-Wha Kang, Sun U. Kwon, Jong S. Kim, Bum Joon Kim

**Affiliations:** 1 Department of Neurology, Asan Medical Center, University of Ulsan, 388-1 Pungnap-Dong, Songpa-Gu, Seoul, 138-736 Korea; 2 Department of Neurology, Gil Medical Center, Gachon University, Incheon, South Korea; 3 Department of Neurology, Hanyang University College of Medicine, Seoul, South Korea; 4 Department of Neurology, Gangneung Asan Hospital, University of Ulsan, Gangneung, Gangwon-Do South Korea

**Keywords:** Cerebrovascular disorders, Stroke

## Abstract

Early neurological deterioration (END) in lenticulostriate artery (LSA) infarction is associated with perforating artery hypoperfusion. As middle cerebral artery (MCA) tortuosity may alter hemodynamics, we investigated the association between MCA tortuosity and END in LSA infarction. We reviewed patients with acute LSA infarction without significant MCA stenosis. END was defined as an increase of ≥ 2 or ≥ 1 in the National Institutes of Health Stroke Scale (NIHSS) total or motor score, respectively, within first 72 h. The MCA tortuosity index (actual /straight length) was measured. Stroke mechanisms were categorized as branch atheromatous disease (BAD; lesions > 10 mm and 4 axial slices) and lipohyalinotic degeneration (LD; lesion smaller than BAD). Factors associated with END in LD and BAD were investigated. END occurred in 104/390 (26.7%) patients. A high MCA tortuosity index (adjusted odds ratio, aOR 10.63, 95% confidence interval [2.57–44.08], p = 0.001) was independently associated with END. In patients with BAD, high initial NIHSS score (aOR 1.40 [1.03–1.89], p = 0.031) and presence of parental artery disease (stenosis < 50%; aOR 10.38 [1.85–58.08], p = 0.008) were associated with END. In patients with LD, high MCA tortuosity (aOR 41.78 [7.37–237.04], p < 0.001) was associated with END. The mechanism causing END in patients with LD and BAD may differ.

## Introduction

Ischemic stroke, classified as small vessel occlusive disease (SVO), is caused by occlusion of a single perforating artery, and shows more favorable outcomes than strokes involving other mechanisms^1^. Nevertheless, 20–40% of these patients develop early neurological deterioration (END), which is adversely associated with long-term functional outcomes^2^.

END in SVO has been associated with the location or shape of the ischemic lesion, initial symptom severity, presence of parent artery stenosis, and hypoperfusion^3^. Hemodynamic insufficiency likely plays a critical role in END among patients with SVO, leading to lesion progression^4,5^. Moreover, tortuosity of the parental artery can influence the morphology of the perforator, and the hemodynamics inside the parental artery affect perfusion through the perforators, resulting in progression of the neurological deficit^6^. However, the relationship between middle cerebral artery (MCA) tortuosity and END in patients with lenticulostriate artery (LSA) infarction has not been investigated.

SVO is classified based on two different mechanisms: branch atheromatous disease (BAD), which involves obliteration of the orifice of the perforating arteries by atheroma of the parental artery, and lipohyalinotic disease (LD), which is associated with a disease of the perforating artery itself^1^. Our previous findings showed that high MCA tortuosity was associated with pathogenesis of atherosclerosis, but recently we also found that it was more related to LD than BAD when analyzing the effect of tortuosity according to the SVO mechanisms^1^. The present study aimed to identify the association between MCA tortuosity and END in patients with LSA territory infarction, with consideration of the underlying SVO mechanisms.

## Results

During the study period, 3805 patients were admitted to our stroke center and 390 (10.2%) patients were diagnosed with acute ischemic stroke due to SVO in the LSA territory. The mean age of patients was 67 ± 12 years, 234 (54.5%) patients were men, and the median initial NIHSS score was 3 (1–5). Among these patients, 257 (65.9%) were diagnosed with LD and 133 (34.1%) with BAD. END occurred in 104 (26.7%) patients with 63 (24.5%) cases attributed to LD and 41 (30.8%) cases linked to BAD.

### Factors associated with END

Table 1 shows the baseline characteristics of patients with and without END. Compared to patients without END, those with END were older (69 ± 12 vs. 66 ± 12 years; p = 0.022), more often had hypertension (81.7 vs. 71.7%; p = 0.044), parental artery disease (26.0 vs. 15.4%; p = 0.017), and S-shaped MCAs (50.0 vs. 32.2%; p = 0.001). The initial NIHSS score [4 (2–6) vs. 3 (1–5); p = 0.027) and the MCA tortuosity index (1.22 ± 0.17 vs. 1.16 ± 0.16; p = 0.001) were higher in those with END than in those without END.

**Table 1:** Baseline characteristics of patients with and without early neurological deterioration.

	END− (n = 286)	END+ (n = 104)	*P*-value
Age (years)	66 ± 12	69 ± 12	0.022
Male	177 (61.9)	57 (54.8)	0.207
Hypertension	205 (71.7)	85 (81.7)	0.044
Diabetes mellitus	83 (29.0)	31 (29.8)	0.880
Hyperlipidemia	138 (48.3)	46 (44.2)	0.482
Smoking history	122 (42.7)	37 (35.6)	0.208
Previous stroke history	72 (25.2)	35 (33.7)	0.097
Previous antiplatelet	30 (28.6)	14 (32.6)	0.630
Previous statin	39 (37.9)	13 (30.2)	0.380
Initial NIHSS score	3 (1–5)	4 (2–6)	0.027
Stroke mechanism			0.181
BAD	92 (32.2)	41 (39.4)	
LD	194 (67.8)	63 (60.6)	
White matter hyperintensities			
Periventricular white matter			0.587
0	91 (31.8)	34 (32.7)	
1	105 (36.7)	34 (32.7)	
2	45 (15.7)	22 (21.2)	
3	45 (15.7)	14 (13.5)	
Deep white matter			0.231
Grade 0–1	151 (53.0)	47 (46.1)	
Grade 2–3	134 (47.0)	55 (53.9)	
Microbleeds	64 (23.2)	23 (22.8)	0.932
Lacunes	107 (37.5)	39 (38.2)	0.902
Stenosis degree			0.017
No stenosis	242 (84.6)	77 (74.0)	
Mild stenosis	44 (15.4)	27 (26.0)	
Tortuosity index	1.16 ± 0.16	1.22 ± 0.17	0.001
MCA shape			0.005
Straight	93 (32.5)	24 (23.1)	
C-shape	101 (35.3)	28 (26.9)	
S-shape	92 (32.2)	52 (50.0)	
Discharge NIHSS score	2 (0–4)	6 (4–7)	< 0.001
Poor outcome at 3 months	32 (14.6)	28 (37.3)	< 0.001

Results presented as number (%) or mean ± standard deviation or interquartile range.

*END* Early neurological deterioration, *BAD* branch atheromatous disease, *LD* lipohyalinotic degeneration, *NIHSS* National Institutes of Health Stroke Scale, *MCA* middle cerebral artery.

Presence of hypertension, parental artery disease, S-shaped MCA, severe initial NIHSS score, and a high MCA tortuosity index were associated with END. Multivariable analysis showed that a high MCA tortuosity index (adjusted odds ratio [aOR] 10.63; 95% confidence interval [CI] 2.57–44.08; p = 0.001) was independently associated with END in patients with LSA territory infarction (Table 2).

**Table 2:** Factors associated with early neurological deterioration.

	Unadjusted univariate analysis		Adjusted multivariate analysis*	
	OR (95% CI)	*P*-value	OR (95% CI)	*P*-value
Age (years)	1.02 (1.00–1.04)	0.054	–	
Male	0.75 (0.47–1.18)	0.208	–	
Hypertension	1.77 (1.01–3.09)	0.046	1.65 (0.92–2.93)	0.091
Diabetes mellitus	1.04 (0.64–1.70)	0.880		
Hyperlipidemia	0.85 (0.54–1.34)	0.482		
Smoking history	0.74 (0.47–1.18)	0.209		
Previous stroke history	1.51 (0.93 –2.45)	0.098	–	
Previous antiplatelet	1.21 (0.56–2.60)	0.630		
Previous statin	0.71 (0.33–1.53)	0.381		
Initial NIHSS	1.12 (1.03–1.22)	0.012	1.10 (0.99–1.21)	0.065
White matter hyperintensities				
Periventricular white matter				
0	1 (reference)			
1	0.87 (0.499—1.505)	0.611		
2	1.31 (0.687—2.492)	0.413		
3	0.83 (0.406—1.707)	0.617		
Deep white Matter				
0–1	1 (Reference)			
2–3	1.32 (0.84–2.08)	0.232		
Microbleeds	0.98 (0.57–1.68)	0.932		
Lacunes	1.03 (0.65–1.64)	0.902		
Stroke mechanism				
LD	1 (Reference)			
BAD	1.37 (0.86–2.19)	0.182		
Mild stenosis	1.93 (1.12–3.32)	0.018	1.80 (0.93–3.31)	0.057
Tortuosity index	9.75 (2.50–38.07)	0.001	10.63 (2.57–44.08)	0.001
MCA shape				
Straight	1 (Reference)			
C-shape	1.07 (0.58–1.98)	0.819		
S-shape	2.19 (1.25–3.85)	0.006		

Results are presented as odds ratio (OR) and 95% confidence intervals (CIs).

*BAD* branch atheromatous disease, *LD* lipohyalinotic degeneration, *NIHSS* National Institutes of Health Stroke Scale, *MCA* middle cerebral artery.

*Multivariate logistic regression adjusted for age, sex, initial NIHSS score, hypertension, mild stenosis, previous stroke history, and tortuosity index.

### Factors associated with END according to the stroke mechanisms

Patients with LD more often had hypertension (77.8 vs. 67.7%; p = 0.030), moderate to severe white matter hyperintensities (53.3 vs. 40.0%; p = 0.013), cerebral microbleeds (26.6 vs. 16.0%; p = 0.022), lacunes (42.0 vs. 29.2%; p = 0.014), no MCA stenosis (93.8 vs. 58.6%; p < 0.001), S-shaped MCA (41.6 vs. 27.8%; p = 0.027), and a high MCA tortuosity index (1.19 ± 0.17 vs. 1.16 ± 0.15; p = 0.045) than those with BAD. The initial and discharge NIHSS scores (2 [1–5] vs. 4 [2–6]; p < 0.001, and 2 [1–4] vs. 4 [2–6]; p < 0.001) and the proportion of those with poor outcomes at 3 months (15.5 vs. 31.8%; p = 0.002) were lower in patients with LD than in those with BAD. (Table 3).

**Table 3:** Baseline characteristics of patients according to the stroke mechanisms.

	LD (n = 257)	BAD (n = 133)	*P-value*
Age (years)	67 ± 12	66 ± 12	0.772
Male	156 (60.7)	78 (58.6)	0.695
Hypertension	200 (77.8)	90 (67.7)	0.030
Diabetes mellitus	80 (31.1)	34 (25.6)	0.252
Hyperlipidemia	118 (45.9)	66 (49.6)	0.487
Smoking history	104 (40.5)	55 (41.4)	0.866
Previous stroke history	77 (30.0)	30 (22.6)	0.120
Previous antiplatelet	28 (28.9)	16 (31.4)	0.751
Previous statin	29 (30.5)	23 (45.1)	0.080
Initial NIHSS score	2 (1–5)	4 (2–6)	< 0.001
White matter hyperintensities			
Periventricular white matter			0.301
0	75 (29.2)	50 (37.6)	
1	92 (35.8)	47 (35.3)	
2	48 (18.7)	19 (14.3)	
3	42 (16.3)	17 (12.8)	
Deep white matter			0.013
Grade 0–1	120 (46.7)	78 (60.0)	
Grade 2–3	137 (53.3)	52 (40.0)	
Microbleeds	87 (26.6)	20 (16.0)	0.022
Lacunes	108 (42.0)	38 (29.2)	0.014
Stenosis degree			< 0.001
No stenosis	241 (93.8)	78 (58.6)	
< 50% stenosis	16 (6.2)	55 (41.4)	
Tortuosity index	1.19 ± 0.17	1.16 ± 0.15	0.045
MCA shape			0.027
Straight	72 (28.0)	45 (33.8)	
C-shape	78 (30.4)	51 (38.3)	
S-shape	107 (41.6)	37 (27.8)	
Discharge NIHSS score	2 (1–4)	4 (2–6)	< 0.001
END	63 (24.5)	41 (30.8)	0.181
Poor outcome at 3 months	32 (15.5)	28 (31.8)	0.002

Results are presented as number (%) or mean ± SD or IQR.

*END* Early neurological deterioration, *BAD* branch atheromatous disease, *LD* lipohyalinotic degeneration, *NIHSS* National Institutes of Health Stroke Scale, *MCA* middle cerebral artery.

Although the percentage of patients with END (24.5% vs. 30.8%; p = 0.181) was similar, predictors of END differed between the LD and BAD subgroups. In patients with LD, a high MCA tortuosity index and S-shaped MCA were associated with END. In multivariable analysis, only a high MCA tortuosity index (aOR 41.78; 95% CI 7.37–237.04; p < 0.001) was independently associated with END in patients with LD (Table 4). In contrast, in patients with BAD, a high initial NIHSS score, and the presence of parental artery disease were associated with END. In multivariable analysis, a high initial NIHSS score (aOR 1.40; 95% CI 1.03–1.89; p = 0.031) and the presence of parental artery disease (aOR 10.38; 95% CI 1.85–58.08; p = 0.008) were independently associated with END in patients with BAD (Table 5). Nevertheless, there was no significant interaction between tortuosity index and stroke mechanism on END (p for interaction = 0.367).

**Table 4:** Factors associated with early neurological deterioration in patients with lipohyalinotic degeneration.

Factors	Univariable analysis		Multivariable analysis*	
	OR (95% CI)	*P*-value	OR (95% CI)	*P*-value
Age (years)	1.02 (0.99–1.04)	0.160	–	
Male	0.69 (0.39–1.23)	0.209	–	
Hypertension	1.70 (0.80–3.59)	0.169		
Diabetes mellitus	0.77 (0.41–1.45)	0.414		
Hyperlipidemia	0.78 (0.44–1.39)	0.395		
Smoking history	0.67 (0.37–1.21)	0.186		
Previous stroke history	1.49 (0.82–2.72)	0.193		
Previous antiplatelet	0.67 (0.24–1.89)	0.448		
Previous statin	0.60 (0.21–1.70)	0.336		
Initial NIHSS score	1.07 (0.95–1.21)	0.264	–	
White matter hyperintensities				
Periventricular white matter				
0	1 (Reference)			
1	0.76 (0.375–1.557)	0.459		
2	1.25 (0.564–2.772)	0.583		
3	0.65 (0.257–1.631)	0.356		
Deep white Matter				
Grade 0–1	1 (Reference)			
Grade 2–3	1.23 (0.69–2.18)	0.483		
Microbleeds	0.92 (0.48–1.77)	0.805		
Lacunes	0.88 (0.49–1.57)	0.665		
Mild stenosis	0.70 (0.19–2.53)	0.582		
Tortuosity index	41.78 (7.37–237.04)	< 0.001	41.78 (7.37–237.04)	< 0.001
MCA shape				
Straight	1 (Reference)			
C-shape	1.24 (0.51–3.03)	0.638		
S-shape	3.70 (1.71–8.03)	0.001		

Results are presented as odds ratio (OR) and 95% confidence intervals (CIs).

*NIHSS* National Institutes of Health Stroke Scale, *MCA* middle cerebral artery.

*Multivariable logistic regression adjusted for age, sex, initial NIHSS score, and tortuosity index.

## Discussion

In the present study, the proportion of patients with END was 26.7% in patients with LSA territory infarction, which was consistent with the results of previous studies^2,3,7^. We found that high MCA tortuosity was independently associated with END. In particular, subgroup analysis of patients with LD showed that high MCA tortuosity was significantly associated with END, whereas the presence of parental artery disease was independently associated with END in patients with BAD (Table 5).

**Table 5:** Factors associated with early neurological deterioration in patients with branch atheromatous disease.

Factors	Univariable analysis		Multivariable analysis*	
	OR (95% CI)	*P*-value	OR (95% CI)	*P*-value
Age (years)	1.02 (0.99–1.06)	0.167	–	
Male	0.86 (0.41–1.81)	0.690	–	
Hypertension	2.08 (0.89–4.89)	0.091	–	
Diabetes mellitus	1.87 (0.83–4.21)	0.133		
Hyperlipidemia	0.95 (0.46–1.99)	0.897		
Smoking history	0.87 (0.41–1.84)	0.716		
Previous stroke history	1.70 (0.73–3.97)	0.219		
Previous antiplatelet	2.89 (0.84–9.97)	0.093	4.04 (0.76–21.44)	0.101
Previous statin	0.79 (0.24–2.56)	0.691		
Initial NIHSS score	1.58 (1.01–1.33)	0.040	1.40 (1.03–1.89)	0.031
White matter hyperintensities				
Periventricular white matter				
0	1 (Reference)			
1	1.09 (0.453–2.626)	0.846		
2	1.50 (0.490–4.588)	0.477		
3	1.40 (0.435–4.522)	0.571		
Deep white matter				
0–1	1 (Reference)			
2–3	1.67 (0.78–3.57)	0.186		
Microbleeds				
Lacunes	1.56 (0.70–3.49)	0.276		
Mild stenosis	2.87 (1.30–5.92)	0.008	10.38 (1.85–58.08)	0.008
Tortuosity index	0.79 (0.07–8.95)	0.848		
MCA shape				
Straight	1 (Reference)			
C-shape	0.92 (0.39–2.21)	0.856		
S-shape	1.06 (0.42–2.70)	0.898		

Results are presented as odds ratio (OR) and 95% confidence intervals (CIs).

*NIHSS* National Institutes of Health Stroke Scale, *MCA* middle cerebral artery.

*Multivariable logistic regression adjusted for age, sex, initial NIHSS score, hypertension, previous antiplatelet, and mild stenosis.

The mechanism associated with END in patients with LSA infarction is relatively homogenous and mainly involves growth of the infarction at the area of diffusion–perfusion mismatch^4,5,8^. As the perforator is an end-artery, infarct growth is dependent on the flow through the perforator rather than through collaterals^9^. END was not rare in this population in the acute stage^2^. The proportion of patients showing END was still considerable among patients with LD. Although the LD pathology itself is stable, flow through the LSA in the acute stage of stroke may be influenced by arterial pressure, blood viscosity, and the tortuosity of the MCA and may lead to END^3,10^.

We have previously shown that the occurrence of LD in LSA territory was associated with a more tortuous shape of MCA^1^. Our current finding that the progression of LSA territory infarction with LD in the acute stage of stroke may be associated with MCA tortuosity can be explainable based on several hypotheses. First, tortuosity of a blood vessel is a systemic problem and the perforator itself may also show high tortuosity^11^. Perforators with a higher tortuosity may have a length longer than average, which is associated with END^12^. Second, matrix metalloproteinase (MMP) is associated with flow-induced vascular remodeling, resulting in high vascular tortuosity^13,14^. The acute inflammatory response regulated by MMP is also associated with the neurological worsening after acute lacunar infarction^15,16^. Third, the distortion and stretching of the perforators by the tortuous MCA may decrease the perfusion through the LSA, predisposing to END^6,10^. Finally, the tortuosity of the parent artery may affect the local hemodynamics and induce local turbulence^17,18^. Local turbulence may reduce the perfusion through the perpendicularly branching perforators^6,10^.

Contrarily, in patients with BAD, presence of parental artery disease was associated with END, which was consistent with the findings of a previous report^2^. This was explained by the atherosclerosis in the parent artery that blocked the orifice of the perforating artery^2^. High arterial tortuosity also enhances the progression of atheroma stemming from the low shear stress area distal to the atheroma^17,18^. However, in cases of BAD, the presence of atheroma in the parental artery, rather than the tortuosity, may be the rate-limiting factor influencing the flow through the perforator. Also, as high tortuosity index was significantly associated with END in overall patients, the relatively low tortuosity index in BAD patients may partially explain the lack of association between the tortuosity index and END.

Until recently, predictive factors associated with END were rarely studied in patients with specific mechanism of LD. Most reports have focused on the factors associated with END in patients with SVO, but not according to its detail mechanisms^4,5,7,8,10,19^. Based on the fact that END in LSA infarction leads to a high rate of functional disability, early identification of patients at risk of progression may improve their clinical and therapeutic management.

Our study has several limitations. First, the number of stroke cases was small, and the study was performed in a single center. Second, the diagnosis of BAD in the present study was defined using conventional MRI, which may not be sensitive enough to distinguish BAD from LD. A study with high-resolution vessel wall MRI may be helpful in distinguishing BAD from LD in future. Also, there is no standard etiological classification based on infarct morphology nor has this classification system been examined in large cohorts. Therefore, we additionally analyzed single subcortical infarction (SSIs) into 3 categories: SSI associated with parent artery disease (SSI-PAD), arteriosclerotic proximal perforator disease without PAD (pSSI-PAD), and distal perforator disease without PAD (dSSI-PAD)^20^. We found that high MCA tortuosity index was independently associated with distal perforator disease without PAD, which was the similar result from the present study. (Supplementary Tables 1 and 2) Third, vascular geometry data were based on a two-dimensional image of a three-dimensional reconstructed TOF-MRA. Recently, various methods involving automated high-performance techniques have been introduced to measure vascular tortuosity in three dimensions^17^. However, the inter-rater reliability of our method was fairly good (Cronbach’s alpha: 0.799). Additionally, the simpler method of categorizing based on vessel shape can be more easily used in clinical practice. Finally, although we suggest an impaired hemodynamic status as one of the mechanisms of END, these issues could not be assessed in this retrospective study. Finally, although we suggest an impaired hemodynamic status as one of the mechanisms of END, these issues could not be assessed in this retrospective study due to the absence of perfusion imaging. Also, to achieve a more comprehensive relationship between atherosclerosis development and the specific tortuosity of perforating arteries and END, future prospective studies employing high-resolution MRI may be necessary.

Despite the limitations of our study, our results suggest that high MCA tortuosity may be a factor associated with END, particularly in cases of SVO involving LD mechanism. Furthermore, the mechanism underlying END in cases with SVO related to LD and BAD may differ. As END in patients with LSA infarction leads to a high functional disability rate, early identification of patients who are at risk of progression may improve their clinical and therapeutic management.

## Methods

### Participants

We retrospectively reviewed patients with acute (< 7 days after stroke onset) LSA territory infarctions, confirmed by magnetic resonance imaging (MRI), who were admitted to the Asan Medical Center from January 2018 to June 2022. Patients were included in this study if they had isolated single small subcortical infarcts of no greater than 20 mm in diameter located within the territories of the LSA^21^ and if they showed no or mild stenosis (< 50%) of MCA on magnetic resonance angiography (MRA). We excluded patients who had any of the following: (1) a significant (> 50%) stenosis of the corresponding extracranial or intracranial artery; (2) any potential causes of embolisms (i.e., embolic heart disease or coagulopathy), (3) other known causes of intracranial stenosis, regardless of the degree of stenosis (i.e., Moyamoya disease or intracranial arterial dissection).

The local ethics committee of Asan Medical Center, South Korea, approved this study (IRB number: 2021-1879). The need to obtain informed patient consent was waived due to the retrospective nature of the study. All methods of this study were performed following the relevant guidelines and regulations.

### Clinical data and END

Demographic data and risk factors were obtained by reviewing medical records and stroke registry database records. Hypertension was defined as receiving medication for hypertension or blood pressure > 140/90 mmHg on repeated measurements. Diabetes mellitus was defined as receiving medication for diabetes mellitus, fasting blood sugar ≥ 126 mg/dL, or 2-h postprandial blood sugar ≥ 200 mg/dL. Hyperlipidemia was defined as overnight fasting cholesterol level > 200 mg/dL or LDL ≥ 130 mg/dL. Previous stroke history was defined as history of stroke at least one diagnosed with a stroke by a physician. History of smoking was defined as people who had smoked before the current hospitalization episode, including current smokers and former smokers. The neurological deficit associated with stroke was evaluated using the National Institute of Health Stroke Scale (NIHSS) score at admission and at discharge.

END was defined as an increase, within the first 72 h of admission of ≥ 2 in the NIHSS total score or of ≥ 1 in the NIHSS motor score^10^, which was not considered to be caused by non-neurological conditions, such as worsening of the medical condition or body injury. The functional outcome was measured by using the modified Rankin scale score at 3 months after discharge and was categorized as good (score: 0–2) or poor (score: 3–6) by a stroke neurologist, either during the patients’ outpatient visits or by telephone.

### Stroke mechanisms

**Figure 1:**
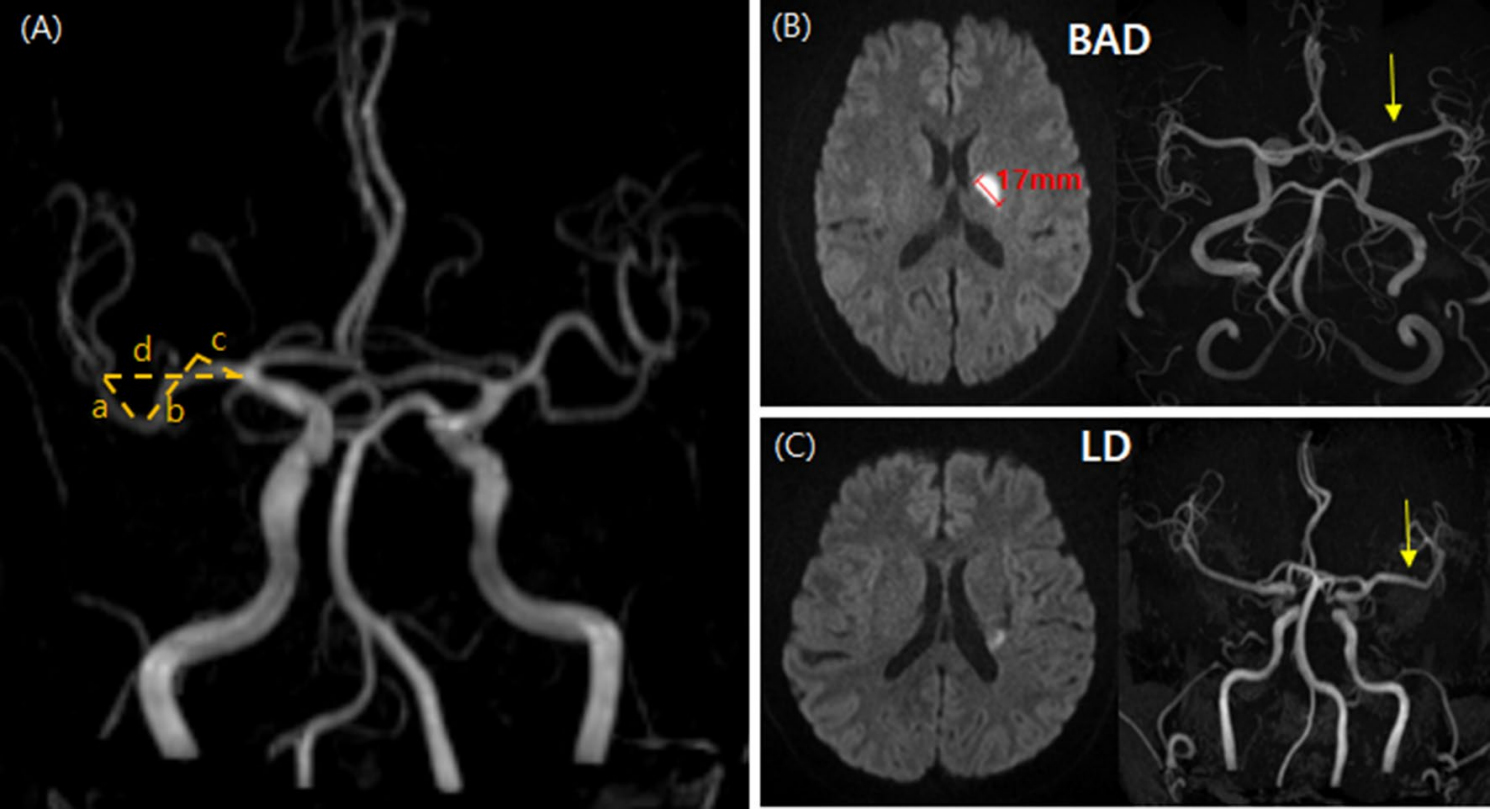
(**A**) Measuring of the MCA TI. The actual length of the MCA is (a + b + c), and the straight length is d. The TI was calculated as ([a + b + c]/d); BAD (**B**) and LD (**C**). *MCA* Middle cerebral artery, *TI* Tortuosity index, *BAD* Branch atheromatous disease, *LD* lipohyalinotic degeneration.

BAD was defined as infarcts caused by occlusion of the orifices or proximal portions of penetrating arteries. Based on diffusion-weighted imaging, BAD of the LSA was defined as infarcts > 10 mm in diameter on the axial slice and visible on four or more axial slices at a slice thickness of 7 mm^21^. Infarcts smaller than this were classified as LD^21^ (Fig. 1B,C).

### Imaging data and tortuosity

White matter lesions were defined as periventricular and deep white matter, and each region were rated using both the modified Fazekas scale. In periventricular white matter, severity was graded as 0 = absent; 1 = pencil-thin lining; 2 = halo of ≥ 5-mm thickness; 3 = irregular white matter hyperintensities extending into the deep white matter. In deep white matter, the severity was graded as 0 = absent, 1 = punctate foci, 2 = beginning confluence, 3 = large confluent areas, which was dichotomized into two groups: grades 0–1 and 2–3^22^. Cerebral microbleeds (CMBs) were identified as small perivascular hemosiderin deposits, which could be visualized as small, rounded, homogeneous, and hypointense lesions on T2*-weighed gradient-recalled echo or susceptibility-weighted images. For this study, all CMBs that had deep and lobar locations were included. Lacune was defined as a round or ovoid, subcortical, fluid-filled (similar signal as CSF) cavity, of between 3 mm and about 15 mm in diameter, consistent with a previous acute small deep brain infarct or hemorrhage in the territory of one perforating arteriole^23^.

Stenosis of the intracranial artery was evaluated based on the time-of-flight (TOF)-MRA. Parental artery disease was defined as a signal reduction of < 50% of the nearest normal sized vessel by referring to a prior segment of vessel without stenosis^24^. The MCA tortuosity index was calculated as actual length (a + b + c) /straight length (d) that two lines starting from ACA-MCA bifurcation point and MCA bifurcation point and running through the midline of MCA were drawn^18^ (Fig. 1A). We also evaluated the shapes of symptomatic MCAs because it may assist in the intuitive understanding of vascular tortuosity. According to our previous report, MCA shapes were classified into three groups: (1) straight; (2) a quadratic curve (single angulation; U-shaped MCA), and (3) a cubic curve (double angulation; S-shaped MCA)^1^.

### Statistical analysis

The baseline characteristics in patients with and without END were compared. Chi-squared or Fisher’s exact tests were used to compare categorical variables, and Student’s *t-*tests or Mann–Whitney U-tests were used to compare continuous variables. Univariable and multivariable analyses were performed to investigate the factors associated with END. According to the results of univariate analyses, age, sex (male), and variables with an associated p value < 0.10 were included in the multivariable logistic regression analysis.

In this study, we further investigated the differences in END-predictive factors according to the stroke mechanisms. For this, we performed univariable and multivariable analyses to evaluate the factors associated with END in subgroups of patients with two different stroke mechanisms – BAD and LD, respectively. Age, sex, and variables yielding a p value < 0.10 in univariate analysis were analyzed using multivariable logistic regression analysis. IBM SPSS version 21.0 software (SPSS, Chicago, IL) was used for all analyses and p values < 0.05 were considered statistically significant.

### Ethical approval

The local ethics committee, ASAN medical center, South Korea, approved this study (IRB number: 2021-1879).

### Informed consent

Due to retrospective nature of the study, need for informed consent was waived by Institutional Review Board of Asan medical center.

### Supplementary Information

Supplementary Tables.

## Data Availability

The data that support the findings of this study are available from the corresponding author on reasonable request.
