# Supplementary material for: Association between arterial tortuosity and early neurological deterioration in lenticulostriate artery infarction
Source: Sci Rep. 2023 Nov 14;13:19865. doi: 10.1038/s41598-023-47281-8 (PMC10646100; doi:10.1038/s41598-023-47281-8)
Supplement: Supplementary file 1 — Supplementary Tables. [file 41598_2023_47281_MOESM1_ESM.docx]

**Supplementary Table 1. Baseline characteristics of patients according to the stroke mechanisms**

|  | **Distal perforator disease without PAD (n=241)** | **Proximal SSI-without PAD (n=78)** | **SSI with PAD (n=71)** | ***P -value*** |
| --- | --- | --- | --- | --- |
| Age (years) | 67 ± 12 | 66 ± 11 | 67 ± 12 | 0.666 |
| Male | 149 (61.8) | 49 (62.8) | 36 (50.7) | 0.207 |
| Hypertension | 186 (77.2) | 52 (66.7) | 52 (73.2) | 0.176 |
| Diabetes mellitus | 76 (31.5) | 20 (25.6) | 18 (25.4) | 0.445 |
| Hyperlipidemia | 109 (45.2) | 39 (50.0) | 36 (50.7) | 0.615 |
| Smoking history | 102 (42.3) | 34 (43.6) | 23 (32.4) | 0.278 |
| Previous stroke history | 70 (29.0) | 15 (19.2 | 22 (31.0) | 0.183 |
| Previous antiplatelet | 27 (29.0) | 11 (30.6) | 6 (31.6) | 0.968 |
| Previous statin | 29 (31.9) | 16 (44.4) | 7 (36.8) | 0.408 |
| Initial NIHSS score | 2 (1 – 5) | 3 (2 - 5) | 5 (3 – 7) | <0.001 |
| **White matter hyperintensities** |  |  |  | 0.07 |
| Grade 0–1 | 114 (47.3) | 48 (62.3) | 36 (52.2) |  |
| Grade 2–3 | 127 (52.7) | 29 (37.7) | 33 (47.8) |  |
| Microbleeds | 62 (26.3) | 10 (13.2) | 15 (23.1) | 0.062 |
| Lacunes | 103 (42.7) | 22 (28.6) | 21 (30.4) | 0.032 |
| **Tortuosity index** | 1.18 ± 0.17 | 1.17 ± 0.16 | 1.14 ± 0.14 | 0.179 |
| **MCA shape** |  |  |  | 0.285 |
| Straight | 67 (27.8) | 27 (34.6) | 23 (32.4) |  |
| C-shape | 76 (31.5) | 30 (38.5) | 23 (32.4) |  |
| S-shape | 98 (40.7) | 21 (26.9) | 25 (35.2) |  |
| **Discharge NIHSS score** | 2 (1 – 4) | 3 (1 – 5) | 5 (2 – 7) | <0.001 |
| **END** | 60 (24.9) | 17 (21.8) | 27 (38.0) | 0.049 |
| **Poor outcome at 3 months** | 30 (15.6) | 14 (25.0) | 16 (34.8) | 0.010 |

Results are presented as number (%) or mean ± SD or IQR

END: Early neurological deterioration, BAD: branch atheromatous disease, LD: lipohyalinotic degeneration; NIHSS: National Institutes of Health Stroke Scale, MCA: middle cerebral artery; SSI: Single subcortical infarction, PAD: Parent artery disease

**Supplementary table 2. Factors associated with END in patients with Distal** **perforator disease without PAD**

| **Factors** | **Univariable analysis** | | **Multivariable analysis ^a^** | |
| --- | --- | --- | --- | --- |
|  | **OR (95% CI)** | ***P-value*** | **OR (95% CI)** | ***P-value*** |
| Age (years) | 1.02 (0.991 – 1.041) | 0.221 |  |  |
| Male | 0.69 (0.379 – 1.238) | 0.210 |  |  |
| Hypertension | 1.93 (0.882 – 4.228) | 0.100 |  |  |
| Diabetes mellitus | 0.82 (0.430 – 1.553) | 0.538 |  |  |
| Hyperlipidemia | 0.83 (0.457 – 1.489) | 0.523 |  |  |
| Smoking history | 0.67 (0.363 – 1.219) | 0.187 |  |  |
| Previous stroke history | 1.45 (0.777 – 2.706) | 0.242 |  |  |
| Previous antiplatelet | 0.56 (0.186 – 1.702) | 0.308 |  |  |
| Previous statin | 0.64 )0.223 – 1.827) | 0.402 |  |  |
| Initial NIHSS score | 1.08 (0.950 – 1.225) | 0.243 |  |  |
| **White matter hyperintensities** |  |  |  |  |
| Grade 0–1 | 1 (Reference) |  |  |  |
| Grade 2–3 | 1.35 (0.751 – 2.444) | 0.314 |  |  |
| Microbleeds | 1.03 (0.529 – 1.996) | 0.936 |  |  |
| Lacunes | 0.86 (0.475 – 1.559) | 0.621 |  |  |
| Tortuosity index | 55.57 (9.264 – 333.38) | <0.001 | 55.57 (9.264 – 333.38) | <0.001 |
| **MCA shape** |  |  |  |  |
| Straight | 1 (Reference) |  |  |  |
| C-shape | 1.52 (0.589 – 3.933) | 0.386 |  |  |
| S-shape | 4.88 (2.10 – 11.314) | <0.001 |  |  |

Results are presented as odds ratio and 95% confidence intervals (CIs).

END: Early neurological deterioration, BAD: branch atheromatous disease, LD: lipohyalinotic degeneration; NIHSS: National Institutes of Health Stroke Scale, MCA: middle cerebral artery, SSI: Single subcortical infarction, PAD: Parent artery disease

^a^ Multivariable logistic regression adjusted for age, sex, initial NIHSS score, and tortuosity index
